# Immunotherapy in gastric cancer—A systematic review

**DOI:** 10.32604/or.2024.052207

**Published:** 2025-01-16

**Authors:** MARTA SANTOS, DIANA MARTINS, FERNANDO MENDES

**Affiliations:** 1 Polytechnic University of Coimbra, ESTESC, UCPCBL, Rua 5 de Outubro, SM Bispo, Apartado, Coimbra, 3046-854, Portugal; 2 H&TRC–Health & Technology Research Center, Coimbra Health School, Polytechnic University of Coimbra, Coimbra, 3046-854, Portugal; 3 Coimbra Institute for Clinical and Biomedical Research (iCBR) Area of Environment Genetics and Oncobiology (CIMAGO), Biophysics Institute of Faculty of Medicine, University of Coimbra, Coimbra, 3000-548, Portugal; 4 Center for Innovative Biomedicine and Biotechnology (CIBB), University of Coimbra, Coimbra, 3000-548, Portugal; 5 European Association of Biomedical Scientists, Brussels, 1000, Belgium

**Keywords:** Gastric neoplasm, Target therapy, Immune checkpoint inhibitors (ICI), Adoptive cell therapy (ACT), Cancer vaccines

## Abstract

**Background:**

Gastric Cancer (GC) is the 5th most prevalent and 4th most deadly neoplasm globally. Immunotherapy has emerged as a promising treatment approach in GC, potentially improving positive clinical outcomes while addressing the limitations of conventional therapies. GC immunotherapy modalities consist of adoptive cell therapy (ACT), cancer vaccines, and immune checkpoint inhibitors (ICI).

**Objectives:**

This systematic review aims to provide an overview of the advances in immune-based therapeutic approaches in GC, highlighting the potential of this therapy as a strategy for GC treatment.

**Methods:**

Key studies investigating several immunotherapeutic agents and combination therapies were searched in PUBMED and included in this study. Specific cancer outcomes related to disease progression or survival were analyzed.

**Results:**

After screening 236 studies, the results revealed that immunotherapy, particularly the ICI pembrolizumab, demonstrated promising efficacy in the treatment of GC, as several studies reported improved OS, PFS, and objective response rate with the use of pembrolizumab alone or in combination with other treatment modalities.

**Conclusion:**

Safety analysis showed that immunotherapy was mostly well-tolerated, with manageable adverse events and relatively good safety profiles. Nonetheless, further research is required to understand the mechanisms of tumor resistance better and identify predictive biomarkers that can direct treatment optimization.

## Introduction

Gastric Cancer (GC) is one of the most common malignancies ranking as the 5th most prevalent and 4th most deadly neoplasm globally [1,2], accounting for approximately 44.0% and 48.6% of new GC cases and GC-related deaths in the world, respectively [3,4]. The incidence rises progressively with age, however, among young adults the number of cases has also been rising [4–6]. Gastric neoplasms can be histologically classified as intestinal, diffuse, or intermediate. Most GC are intestinal type (adenocarcinomas), which are more common in the antral region of the stomach and are sporadic being mainly associated with *Helicobacter pylori* infection [7].

Generally, GC patients are diagnosed at advanced stages of the disease due to the lack of clinical symptoms and efficient screening programs, with a median of overall survival (OS) of approximately 12 months for patients with metastatic disease [8]. Dietary habits, such as the high consumption of spicy, pickled, and salty foods, alcohol and cigarette use, as well as family history of cancer and concomitant infections (namely by *H. pylori*) are the most significant risk factors for developing GC [9].

Diagnosis can be achieved through a biopsy in combination with endoscopic imaging techniques [10,11]. Treatment options are mainly selected based on the stage of the disease. Overall, 60% of people with gastric cancer are not eligible for curative treatment owing to late presentation or comorbidities. The 5-year survival for stage IA and IB tumors treated with surgery (gastrectomy) is between 60% and 80%. However, patients with stage III tumors undergoing surgery, with or without the administration of perioperative chemotherapy have a dismal 5-year survival rate. Relapse rates are high and the five-year survival rate is inferior to 5% [12].

Despite the advances in diagnostic tools in GC, and the importance of targeted therapies, such as the human epidermal growth factor receptor (HER2) [13] and Epidermal Growth factor receptor (EGFR) that demonstrated improvements in overall survival in patients with advanced GC [14], the effects on mortality have been modest. The genetic profiling of gastric cancers and the identification of molecular biomarkers offer the possibility of new and personalized therapeutic targets. *Nuclear Factor Erythoid 2-Related Factor 2 (Nrf2)* is a transcriptor factor that can function as an oncogene or tumor suppressor gene, suggesting that the Nrf2 signaling pathway can be used as a therapeutic target [15]. Sex-determining region Y (Sry)-box-containing (SOX) family members are transcription factors that can play a dual role in cancer. The progression and/or inhibition of GC in SOX pathway members is described, suggesting that can be used as a diagnostic or prognostic biomarker in GC [16]. Resveratrol, a non-flavonoid polyphenol, with anti-tumor properties such as apoptosis, cell proliferation inhibition, and anti-inflammatory responses has also been studied in GC. The regulation of Wnt and PI3K signaling pathways, associated with the ability to sensitize gastric cancer cells to chemotherapy, suggests that Resveratrol can be used as a therapeutic target for GC patients [17]. *Phospathase and tensin homolog (PTEN)* is a tumor-suppressor gene that inhibits the PI3K/Akt signaling pathway, decreasing migration and growth of gastric cancer cells and can inhibit the epithelial-to-mesenchymal transition (EMT) mechanism, suggesting its role as a possible therapeutic target in GC [18].

Conventional treatments for GC are limited in clinical efficacy, and the developing understanding of GC pathogenesis has increased the demand for new therapeutic regimens, including immunotherapy. This type of treatment, along with emerging molecular techniques to characterize the tumor, may be a powerful personalized therapy option for GC patients [11,12].

Cancer immunotherapy aims to restore and induce a powerful immune response, using the host immune system, using its reactivity against tumor cells [13], killing them associated with tumor regression. Furthermore, cancer immunotherapy aims to overcome the evasive strategies used by cancer cells to avoid destruction by immune cells. This requires countering the elusive strategies utilized by tumor cells to circumvent elimination [14]. Therefore, the fundamental theory of immunotherapy relies on the immune system’s ability to be reconditioned, restoring a powerful anti-tumor response [15].

### Adoptive cell therapy

**Figure 1:**
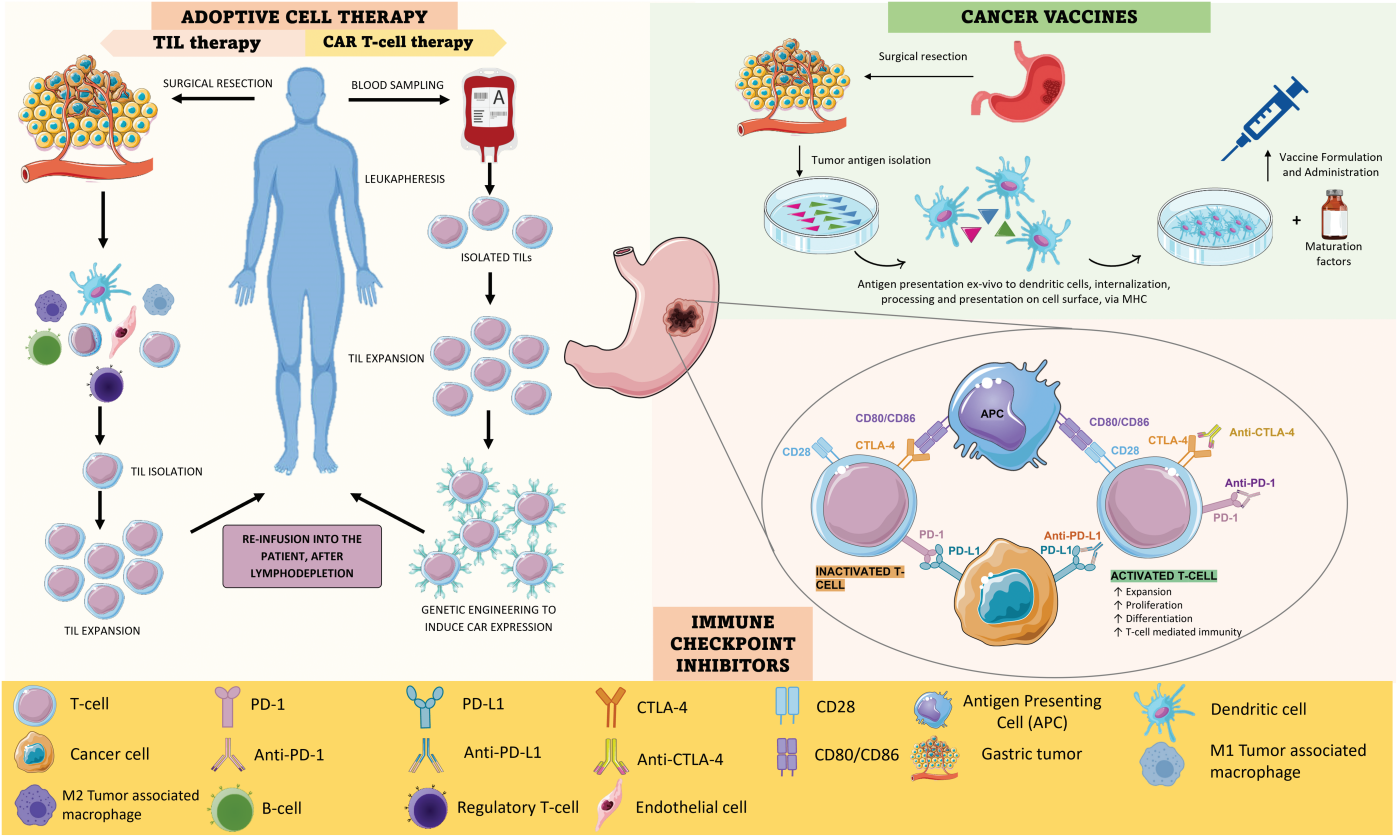
A). ACT strategies: TIL therapy and CAR-T cell therapy. In TIL therapy, lymphocytes that have naturally infiltrated the TME are isolated from resected tumor tissue and expanded *ex vivo*; after lymphodepletion, they are re-administered to the patient, constituting a potent anti-tumor response. In CAR-T cell therapy, lymphocytes are isolated from a peripheric blood sample through leukapheresis and then expanded; after that, the cells are genetically engineered to induce CAR expression, which facilitates cancerous recognition and elimination without the need for MHC-mediated antigen presentation; finally, the genetically modified T-cells are re-infused. B). CANCER VACCINES–There are many types of cancer vaccines, using mRNA, DNA, and dendritic cells. Dendritic cell cancer vaccines have been extensively studied and have shown potential in clinical studies. Their production involves the collection of a tumor sample and the isolation of tumor-specific antigens; then, dendritic cells are exposed to these antigens, internalizing, processing, and expressing them on their surface. After this, these cells are expanded *ex vivo* by being mixed with immune-stimulating substances, formulating the cancer vaccine, which can then be administered to the patient. In the organism, these cells form a robust anti-tumor response (active cancer cases) or prepare the immune system of genetically predisposed individuals to fight off cancer cells, should they ever arise. C). Mechanism of action of anti-PD-1 and anti-CTLA-4. PD-1 is expressed on the surface of T cells and when it binds to PD-L1 it triggers a signaling cascade that leads to T cell inactivation; as such, cancer cells tend to overexpress PD-L1 to down-regulate T-cell mediated responses. Both anti-PD-1 and anti-PD-L1 are antibodies that can inhibit this cascade by binding to PD-1 and PD-L1, respectively, leading to T-cell activation and cancer cell elimination. CTLA-4 on the other hand is a molecule that T cells express that can inhibit T cell proliferation and activity because it binds with higher affinity than CD28 to CD80 and CD86; when CD28 binds to these ligands, it leads to T cell activation, however, if CTLA-4 is present, this does not occur, once cancer cells upregulate CTLA-4 expression. Anti-CTLA4 is an antibody that binds to CTLA-4 and therefore allows CD28 to connect to CD80 and CD86, enhancing T-cell mediated immune responses. Fig. 1 was created using Biorender (https://biorender.com/, accessed on 21 November 2024).

Adoptive Cell Therapy (ACT), or cellular immunotherapy, is a treatment approach that harnesses the host’s immune system’s own cells, educating them to eliminate cancer cells. It comprises two techniques-tumor infiltrating lymphocyte (TIL) therapy and chimeric antigen receptors (CAR)-T cell therapy [12], as observed in Fig. 1A.

TIL are lymphocytes that have naturally infiltrated the tumor stroma, where they can recognize and attack cancer cells, becoming activated. However, they are often present in insufficient numbers to constitute an effective anti-tumor immune response. This technique involves the isolation of the patient’s TIL from resected tumor tissue which, when activated and in sufficient numbers, is powerful in eliminating tumor cells. Their activation and expansion *in vitro*, and later re-infusion into the patient, triggers a robust anti-tumor immune response that results in tumor ablation [19–22].

Since not all cancer patients’ T-cells have already infiltrated the tumor, circulant T-cells may be collected from peripheral blood. This process involves the leukapheresis of the collected sample to isolate T-cells, which are then genetically engineered to enhance their tumor-fighting abilities. The genetic modification is done using viral vectors which introduce the gene encoding the CAR into T-cells, therefore transforming them into CAR-T cells. This receptor makes T-cells recognize TAA and eliminate them [15,17].

Normal T-cell activation is heavily reliant on the presentation of antigens that are bound through the major histocompatibility complex (MHC), which cancer cells normally underexpress. CAR-T therapy allows for the bypassing of MHC-mediated antigen presentation, as CAR-T cells can bind to cancerous cells even in the absence of antigen presentation on the cell surface [18–20]. As such, the immune evasion mechanism by cancer cells is circumvented. The biggest obstruction to this therapy approach is related to shared antigens between cancerous and normal tissue cells, which makes it difficult to select a target antigen that would not be associated with toxicity to healthy tissues [19].

### Cancer vaccines

Cancer vaccines are a type of immunotherapy that comes in the form of a vaccine containing peptides derived from TAA, DNA or messenger RNA (mRNA) coding cancer antigens, whole cancer cells that have been modified to be harmless or dendritic cells that have been harvested and exposed to cancer antigens (Fig. 1B) [21]. Using whole cancer cells presents the advantage of allowing the targeting of multiple cancer antigens at the same time. The recognition of the vaccine components as “non-self” by the immune system, and consequent antigen presentation to T-cells, triggers their expansion as effector T-cells, which then target the antigen-expressing cells, eliminating them and promoting tumor ablation [13].

Cancer vaccines are typically administered after cancer is diagnosed, with the intended purpose of stimulating the immune system to become more reactive towards cancer cells and overcoming the immune-inhibiting mechanisms. However, there is an interest in developing cancer vaccines to use as a preventive measure in individuals who are known to be at high risk of cancer development, for example., due to genetic predisposition. These vaccines would be administered before cancer onset, resulting in a faster and stronger immune response against cancer cells if develop in the body [22].

Although there have been several clinical trials for cancer vaccines for GC with promising results, their efficacy has been limited due to high tumor heterogeneity, low levels of TIL, and an immunosuppressive TME. As such, cancer vaccines are not yet commonly used in clinical practice, excluding the hepatitis B virus (HBV) and human papillomavirus (HPV) preventive vaccines, both of which have been approved by the Food and Drug Administration (FDA) [17].

### Immune checkpoint inhibitors

Immune checkpoints (IC) are proteins that are present on the surface of immune cells and act as immune response regulators, preventing the immune system from becoming overactive. The two most well-studied IC in GC are PD-1 and CTLA-4, as shown in Fig. 1C [22]. PD-1 and CTLA-4 are present in the T-cell surface and, when they bind to their ligands, trigger a cascade of intracellular signals that lead to the inhibition of T-cell recruitment and activity. Although these mechanisms are, in physiological conditions, crucial for down-regulating immune responses and maintaining self-tolerance, GC cells often evolve to upregulate PD-1 and CTLA-4 expression to evade immune detection and destruction [19,23–25].

ICI therapy uses monoclonal antibodies (mAb) against IC, resulting in the blockage of the aforementioned checkpoint pathways [13,26]. This therapy aims to block the events that prevent tumor cells from being targeted by the immune system, instead of targeting cancer cells directly [26–28].

Although several studies have proven the successfulness of ICI as a form of treatment for many cancer types, a critical gap remains in our understanding of its efficacy in GC, particularly in identifying patients most likely to benefit from these treatments. Namely, a major roadblock to the widespread use of ICI in anti-cancer therapy is their dependence on pre-existing TIL. As such, patients with fewer or non-immunogenic tumors may not respond to this treatment option [17].

The success of immunotherapy substantially depends on the TME and its interactions with the immune system. An activated immune phenotype is associated with better survival rates, whereas a state of immune suppression significantly hinders immunotherapy’s effects. As such, the identification of biomarkers related to these interactions holds promise for guiding personalized treatment strategies.

This systematic review aims to provide an overview of the advances in immune-based therapeutic approaches in GC, such as adoptive cell therapy (ACT), immune checkpoint inhibitors, and vaccines, to reveal their eventual potential and promising strategy for GC treatment.

## Materials and Methods

The authors used the Preferred Reporting Items for Systematic Reviews and Meta-Analyses (PRISMA) statement [29] to perform this systematic review, to gauge the role of immunotherapy in GC.

This objective was accomplished by conducting a literature search using the PubMed database. The search exploited the following keywords: “Gastric Cancer”, “Immunotherapy”, “Immune Checkpoint Inhibitors”, “Adoptive Cell Therapy” and “Cancer vaccines”. Table 1 outlines the research methodologies employed, which involved the use of Medical Subject Headings terms, and combinations of keywords to obtain the relevant literature for this review.

**Table 1:** A strategy used to obtain the literature used in this review, by combining the keywords (Medical Subject Headings terms) “Gastric Cancer”, “Immunotherapy”, “Immune Checkpoint Inhibitors”, “Adoptive Cell Therapy” and “Cancer Vaccines”, and using the Boolean operators (And, Or and Not)

	Boolean operators		
Medical subject headings terms	And	Or	Not
Gastric cancer	Immunotherapy, immune checkpoint inhibitors		
Gastric cancer	Immunotherapy, immune checkpoint inhibitors		Adoptive cell therapy, cancer vaccines
Gastric cancer	Immunotherapy, adoptive cell therapy		Immune checkpoint inhibitors, cancer vaccines
Gastric cancer	Immunotherapy, cancer vaccines		Immune checkpoint inhibitors, adoptive cell therapy
Gastric cancer	Immune checkpoint inhibitors, adoptive cell therapy		Cancer vaccines
Gastric cancer	Immune checkpoint inhibitors, cancer vaccines		Adoptive cell therapy

The decision to focus exclusively on the PubMed database for this systematic review was carefully considered and based on several factors. Its extensive coverage of peer-review articles granted access to a wide range of studies relevant to our search question. Given time and resource constraints, we opted for PubMed alone, recognizing its ability to capture most of the relevant literature, despite the potential advantages of searching multiple databases.

**Figure 2:**
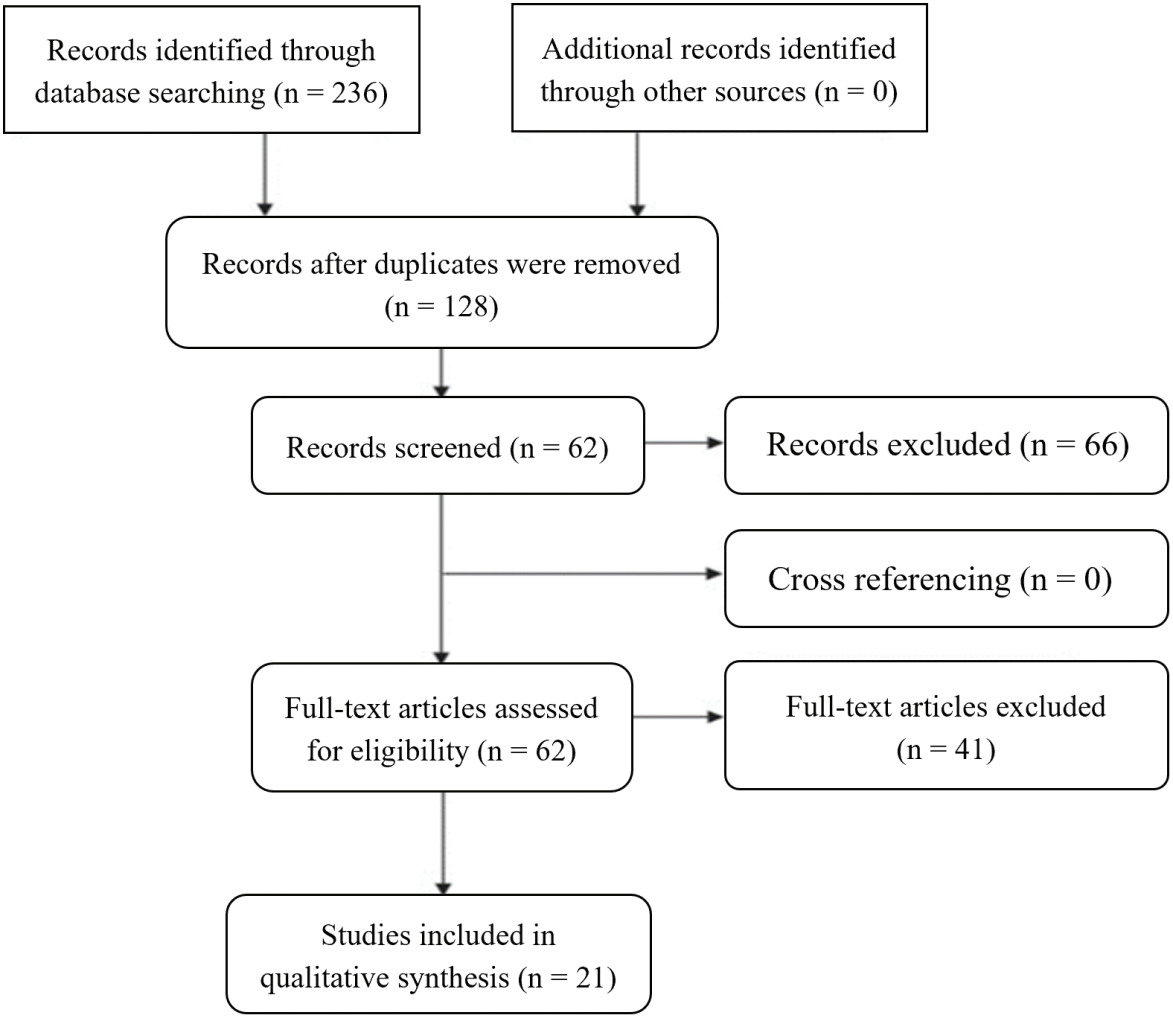
PRISMA systematic, including the literature research performed, the number of articles evaluated, and the total number of articles excluded and included in the review.

The literature research was conducted between April 2022 and May 2023, all articles were identified, analyzed, and selected based on specific inclusion and exclusion criteria. These criteria were designed to ensure that only articles providing pertinent information were included in the study. Free full-text articles published in English between January 2017 and May 2023, including clinical trials, meta-analyses, reviews, and systematic reviews were considered eligible for inclusion. Conversely, any literature that failed to meet these criteria was excluded. Additionally, studies were excluded if they were considered to have limitations such as a high risk of bias, incomplete data reporting, or an unclear study objective, resulting in 21 studies included in this systematic review as described in Fig. 2.

**Figure 3:**
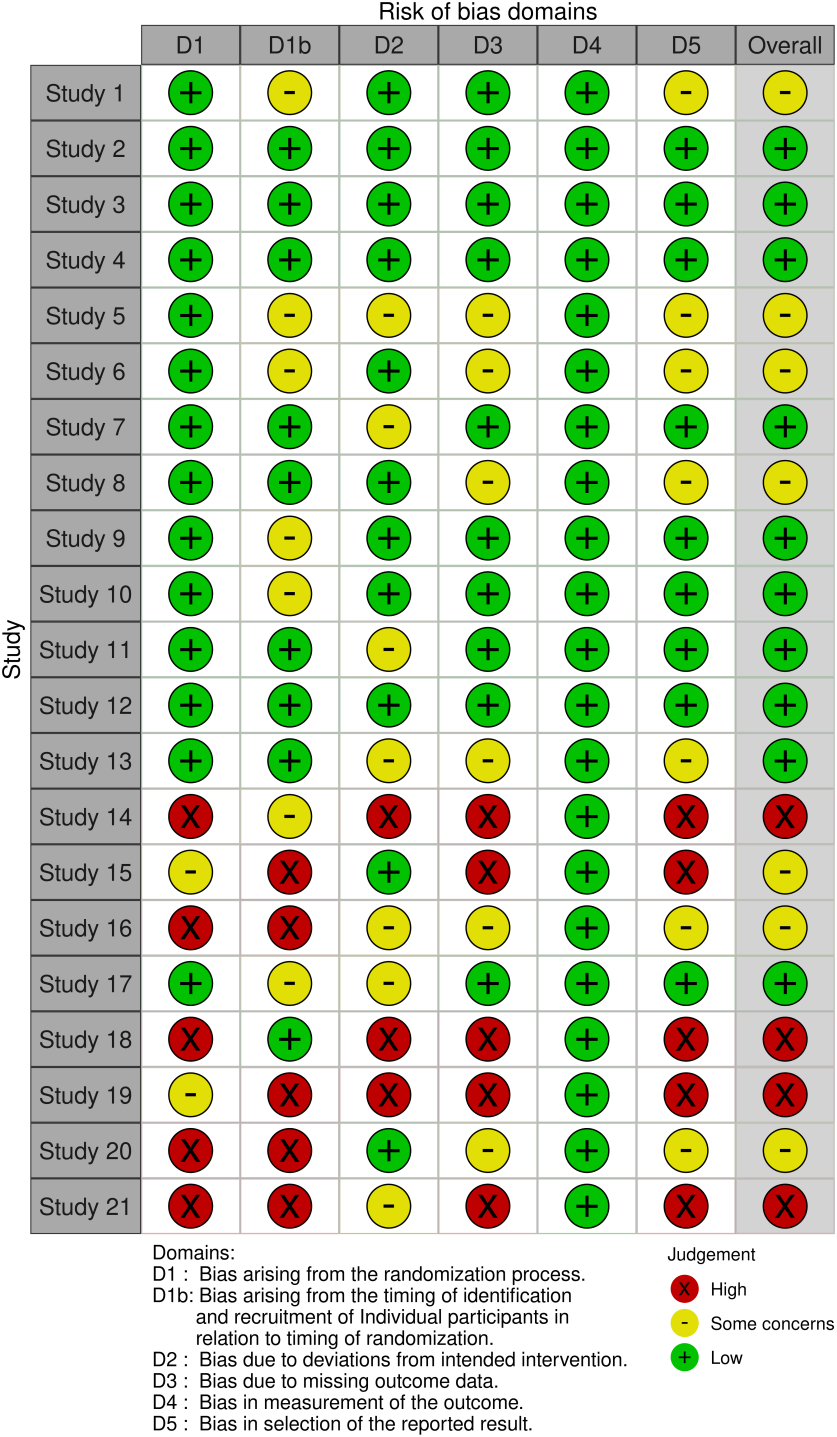
Traffic light plot from the Robvis risk of the bias tool.

The risk of bias assessment for the studies included in this systematic review was conducted using the Robvis tool (Risk-Of-Bias VISualization) (RoB 2), a comprehensive and widely used tool to assess the quality and risk of bias in research studies. The traffic light plot generated by Robvis is represented in Fig. 3, providing a clear and concise overview of the risk of bias assessment of the included studies.

## Results

Advanced-stage and late diagnosis of GC are associated with poor prognosis and limited therapeutic options. Identifying and developing new therapeutic strategies, such as immunotherapy, may contribute to better survival and response rates in GC patients. This review included 21 studies using immunotherapy as a treatment option for GC patients, as observed in Fig. 2.

The KEYNOTE-012, a phase Ib trial was the first time that pembrolizumab, an anti-PD-1 mAb, was used as an immunotherapeutic approach for patients with advanced GC and PD-Ligand 1 (PD-L1) positive (Table 2) [30]. The study included 39 patients, and the overall response rate (ORR) was 22%; the ORR was 50% for patients with high microsatellite instability (MSI-H) tumors, representing 17% of the total population of the study. Only 13% of patients showed grade 3–4 treatment-related adverse effects (AE), including fatigue and hypothyroidism. These promising results drove several studies to assess the safety and efficacy of pembrolizumab, as monotherapy or in combination with chemotherapy for GC patient’s treatment [30].

**Table 2:** The table presents the trial outcomes of recent studies involving ICI (such as pembrolizumab, nivolumab, ipilimumab, trastuzumab, ramucirumab, durvalumab, sintilimab, apatinib and pertuzumab), cancer vaccines and ACT, as well as their combination with different chemotherapies. The effectiveness of the treatments is a direct reflection of their clinical outcomes, namely OS, PFS, and ORR. Additionally, the occurrence of AE is reported to assess the safety profiles of each therapeutic approach/regimen. The results compiled in this table aim to provide a comprehensive overview of the current landscape of immunotherapeutics and therapies for GC, aiding in the understanding of their potential benefits

Study	Antigen target	Drug	Methods	Results	Adverse events
[30] Muro et al., 2016/KEYNOTE-012	PD-1	Pembrolizumab	39 patients received 10 mg/kg of pembrolizumab once every 2 weeks for 2 years	ORR was 22%	13% of patients present with grades 3–4 AE, with the most common being fatigue
[31] Fuchs et al., 2018/KEYNOTE-059	PD-1	Pembrolizumab	259 patients receive 200 mg of pembrolizumab every 3 weeks	ORR was 11.6% [6 CR (2.3%) and 24 PR (9.3%].	17.8% of patients present with grades 3–5 AE, with the most common being fatigue pruritus and rash
				PD-L1 expression (Negative *vs*. positive (CPS ≥ 1): ORR was 15.5% *vs*. 6.4%	
[32] Marabelle et al., 2020/KEYNOTE-158	PD-1	Pembrolizumab	233 patients with MSH-H/dMMR tumors received 200 mg of pembrolizumab every 3 weeks for two years	ORR was 34.3% [23 CR (9.9%) and 57 PR (24.5%]. For GC, the ORR was 45.8 and the median PFS was 11 months	14.6% of patients had 3–5 grade AE, with the most common being fatigue, pruritus, and diarrhea.
[33] Maio et al., 2022 updated results KEYNOTE-158	PD-1	Pembrolizumab	351 patients with various solid tumors with the dMMR MSI-H phenotype (excluding colorectal carcinoma) received Pembrolizumab (200 mg) every 3 weeks for 35 cycles	ORR was 30.8% DOR was 47.5 months. The median PFS was 3.5 months. Median OS was 20.1 months	11.1% of patients had grades 3–4 treatment-related AE, with the most common being myocarditis and pneumonia
[34] Shah et al., 2019/KEYNOTE-180	PD-1	Pembrolizumab	121 patients received 200 mg of pembrolizumab every 3 weeks for up to two years	ORR was 11.6% [6 CR (2.3%) and 24 PR (9.3%].	12.4% of patients presented grades 3–5 AE, with the most common being hypothyroidism and pneumonitis
			Determination of PD-L1 status by immunohistochemistry	PD-L1 expression (Negative *vs*. Positive (CPS ≥ 10): ORR was 13.8% *vs*. 6.3%	
[36] Janjigian et al., 2018/CheckMate-032	PD-1 and CTLA-4	Nivolumab and ipilimumab	160 patients stratified into three different arms:	ORR was 12% (NIVO3) *vs*. 24% (NIVO1+IPI3) *vs*. 8% (NIVO3+IPI1)	17% (NIVO3) *vs*. 47% (NIVO1+IPI3) *vs*. 37% (NIVO3+IPI1) of patients present with grades 3–5 AE, with the most common diarrhea and elevation of liver enzyme levels
			- Nivolumab (NIVO3): 3 mg/kg every two weeks for four cycles		
			- Nivolumab + Ipilimumab (NIVO1+IPI3): nivolumab 1 mg/kg plus ipilimumab 3 mg/kg every two weeks for four cycles	DOR was 7.1 months (NIVO3) *vs*. 7.9 months (NIVO1+IPI3) and not yet reached (NIVO3+IPI1).	
			- Nivolumab + Ipilimumab (NIVO3+IPI1): nivolumab 3 mg/kg plus ipilimumab 1 mg/kg every two weeks for four cycles	12-month OS rate was 39% (NIVO3) *vs*. 39% (NIVO1+IPI3) *vs*. 24% (NIVO3+IPI1)	
[37] Janjigian et al., 2021/CheckMate-649	PD-1	Nivolumab and chemotherapy *vs*. chemotherapy	1549 patients stratified into three different arms:	Median OS was 13.1 months (NIVO+CHEMO) *vs*. 11.1 months (CHEMO)	59% (NIVO+CHEMO) *vs*. 44% (CHEMO) of patients present with grade 3/4 AE, with the most common being nausea, diarrhea and peripheral neuropathy
			- Nivolumab: 360 mg every 3 weeks or 240 mg every 2 weeks) plus-chemotherapy (XELOX every 3 weeks or FOLFOX every 2 weeks) (NIVO+CHEMO)		
			- Chemotherapy alone: XELOX every 3 weeks or FOLFOX every 2 weeks (CHEMO)	Median PFS was 7.7 months (NIVO+CHEMO) *vs*. 6.05 months (CHEMO)	
[38] Janjigian et al., 2021/KEYNOTE-811	PD-1 and HER2	Trastuzumab, chemotherapy and pembrolizumab *vs*. Trastuzumab, chemotherapy and placebo	433 patients stratified into two different arms:	ORR was 74.4% (pembrolizumab) *vs*. 51.9% (placebo) (CR of 11.3% and 3.1%; PR of 63.2% *vs*. 48.9%, respectively)	Approximately 57% of patients in each arm present with grades 3–5 AE, with the most common being diarrhea, nausea and anemia
			Trastuzumab + chemotherapy + Pembrolizumab		
			Trastuzumab + Chemotherapy + Placebo	DCR was 96.2% (pembrolizumab) *vs*. 89.3% (placebo)	
[39] Catenacci et al., 2020/CP-MGAH22-05,	PD-1 and HER2	Margetuximab and pembrolizumab	86 patients received margetuximab (15 mg/kg) and pembrolizumab (200 mg) every 3 weeks	ORR was 18.48%	52% of patients present with grades 3–5 AE, with the most common being anemia and infusion reactions
				DCR was 53%	
				Median PFS was 2.73 months	
				OS was 12.48 months	
[41] Fuchs et al., 2021/KEYNOTE-061	PD-1	Pembrolizumab *vs*. paclitaxel	592 patients stratified into two different arms:	ORR was 15.8% (pembrolizumab) *vs*. 13.6% (paclitaxel)	14.3% (pembrolizumab) *vs*. 34.8% (paclitaxel) of patients present with grade 3/4 AE, with the most common being fatigue and anemia
			-Pembrolizumab: 200 mg every three weeks for up to 35 cycles	OS was 9.1 months (pembrolizumab) *vs*. 8.3 months (paclitaxel)	
			-Chemotherapy: 80 mg/m^2 on days 1, 8, and 15 of 4-week cycles	Median PFS was 1.5 months (pembrolizumab) *vs*. 4.1 months (paclitaxel)	
[41] Fuchs et al., 2022/KEYNOTE-061	PD-1	Pembrolizumab *vs*. paclitaxel	592 patients stratified into two different arms: Pembrolizumab: 200 mg every three weeks for up to 35 cycles	PFS: PD-L1 CPS ≥ 1: Pembrolizumab (1.5 months) *vs*. paclitaxel (4.1 months)	15% (pembrolizumab) *vs*. 35-.1% (paclitaxel) of patients present with grade 3/4 AE, with the most common being fatigue, decreased appetite and nausea
				PD-L1 CPS ≥ 5: Pembrolizumab (1.6 months) *vs*. paclitaxel (4 months)	
			Chemotherapy: 80 mg/m^2 on days 1, 8, and 15 of 4-week cyclesPD-L1 expression assessed using IHQ	PD-L1 CPS ≥ 10: Pembrolizumab similar to paclitaxel (around 2.7 months) ORR: 16.3% (CPS ≥ 1) *vs*. 24.5% (CPS ≥ 10)	
				Median DOR was longer in the pembrolizumab group regardless of CPS status, and increased with higher PD-L1 CPS	
[42] Shitara et al., 2020/KEYNOTE-062	PD-1	Pembrolizumab *vs*. pembrolizumab and chemotherapy *vs*. chemotherapy	763 patients with PD-L1 CPS ≥ 1 were stratified into three different arms:	PD-L1 expression:	70% of patients displayed grades 3–5 AE, with the most common being fatigue, nausea and decreased appetite
			-Pembrolizumab: 200 mg every three weeks	CPS ≥ 1: Similar OS results between pembrolizumab monotherapy and chemotherapy alone	
			-Pembrolizumab (200 mg) plus chemotherapy (cisplatin 80 mg/m^2^/d on day 1 plus fluorouracil 800 mg/m^2^/d on days 1 to 5 or capecitabine 1000 mg/m^2^ twice daily)	• CPS ≥ 10: 17.4 months (pembrolizumab) 10.8 months (chemotherapy)	
			-Chemotherapy (same dosage as the previous group) plus placebo every three weeks		
[43] Kojima et al., 2020/KEYNOTE-181	PD-1	Pembrolizumab *vs*. chemotherapy	628 patients were stratified into two different arms:	PD-L1 positive tumors (CPS ≥ 10)-median OS was 9.3 months (pembrolizumab) *vs*. 6.7 months (chemotherapy)	18.2% (pembrolizumab) *vs*. 40.9% of patients displayed grades 3–5 AE, with the most common being fatigue, hypothyroidism and decreased appetite
			-Pembrolizumab: 200 mg every 3 weeks for up to two years		
			-Chemotherapy: Paclitaxel 80–100 mg/m^2 on days 1, 8, and 15 of each 28-day cycle, docetaxel 75 mg/m^2 on day 1 of each 21-day cycle, or irinotecan 180 mg/m^2 on day 1 of each 14-day cycle	ORR of 21.5% (pembrolizumab) and 6.1% (chemotherapy) survival rate at 12-months was 43% (pembrolizumab) *vs*. 20.4% (chemotherapy)	
			- Determination of PD-L1 status by immunohistochemistry	Median PFS was 2.6 months (pembrolizumab) *vs*. 3.0 months (chemotherapy)	
[44] Bang et al., 2020/JVDJ	PD-1 and VGFR2	Ramucirumab and duravalumab	85 patients were stratified into two different arms:	GC patients showed an ORR of 6%, which amounted to a DCR of 16%. Median PFS was of 2.6 months and median OS of 12.4 months	**For GC/GEJ patients (n = 29)**, 37.9% present with grades 3–5 AE, with the most common being fatigue, hypertension and diarrhea
			- Ramucirumab (10 mg/kg) plus durvalumab (1125 mg) every three weeks		
			-Ramucirumab (8 mg/kg) plus durvalumab (750 mg) every two weeks	PD-L1 expression (High *vs*. low): Median PFS was 14 months *vs*. 12 months; median OS was 14.8 months *vs*. 5.5 months, respectively	
[45] Chung et al., 2019/JAVELIN	PD-L1	Avelumab	150 patients were stratified into two arms that both received 10 mg/kg every 2 weeks:	Second-line (2L):	8.7% of patients in both groups experienced grades 3–5 AE, with the most common being fatigue, anemia and asthenia
				ORR was 6.7%	
				DCR was 28.3	
				Median DOR reached 3.5 months	
			• Second line (2L): chemotherapy more than 28 days prior;	Median PFS was 1.4 months	
				Median OS was 8.6 months	
				First-line maintenance (1L-mn):	
				ORR was 6.7% (2.2% CR and 4.4% PR)	
			First-line maintenance (1L-mn) chemotherapy within 28 days prior;	DCR was 56.7%	
				Median DOR reached 21.4 months	
				Median PFS was 2.8 months	
				Median OS was 11.1 months	
[46] Zhang et al., 2023	VEGFR2 and PD-1	Apatinib, sintilimab and chemotherapy	30 patients were administered sintilimab (200 mg on day one), apatinib (once daily in each cycle) and chemotherapy (paclitaxel or irinotecan; 200 mg every three weeks)	ORR of 53.6% [PR of 53.5% (15 patients), SD of 28.5% (8 patients) and SD of 17.8% (5 patients)]	49.8% of patients present with grades 3–5 AE, with the most common being neutropenia and leukopenia
				DCR was 82.1%	
				Median DOR of 8.8 months	
				Median PFS of 8.5 months	
				Median OS of 12.5 months	
[47] Tabernero et al., 2023/JACOB	HER2	Pertuzumab, trastuzumab and chemotherapy	780 patients were randomized into two arms:	Median OS of 18.1 months (pertuzumab) *vs*. 14.2 (placebo)	80.5% (pertuzumab) *vs*. 74.2% (placebo) of patients present with grades 3–5 AE, with the most common being diarrhea
			- Pertuzumab (840 mg) plus trastuzumab (8 mg/kg and 6 mg/kg maintenance doses) and chemotherapy (intravenous cisplatin 80 mg/m^2^ plus capecitabine 1000 mg/m^2^ twice daily, or intravenous 5-furouracil 800 mg/m^2^ every 24 h continuously for 120 h), every 3 weeks	Median PFS of 8.5 months *vs*. 7.2 months	
			- Pertuzumab plus placebo plus chemotherapy (same dosage)		
[48] Ishikawa et al., 2018	HER2 AND EGFR	Trastuzumab- and cetuximab-based chemotherapy	GC patients received chemotherapy (1000 mg/m^2^ capecitabine or 80 ng/m^2^ S^−1^) for 14 days. After a week rest, they were administered 80 mg/m^2^ cisplatin. 8 mg/kg trastuzumab was administered on day 1 of the first cycle, followed by 6 mg/kg every 3 weeks. Expanded NK cells were injected for 60 min, 3 days after antibody administration, every 3 weeks for 3 cycles	DCR was 66.7% (0% CR, 66.7% SD)	The most common adverse events (anemia, thrombocytopenia, fatigue, and peripheral neuropathy) were observed at similar rates as those reported with standard chemotherapy regimens
[49] Qi et al., 2022/Claudin18.2	CLDN18.2	CT041	49 patients were infused with CT041 for 12 weeks (at the time of interim analysis)	ORR was 48.6%.	100% of patients showed grades 3–4 AE, with the most common hematologic toxicities, leukopenia, neutropenia, and anemia
				DCR was 73%.	
				Median PFS was 3.7 months	
				For GC patients (n = 28):	
				ORR was 57.1%	
				DCR was 75%	
				Median PFS was 4.2 months	
[50] Ede et al., 2022/HER2-vaxx	HER2 and VEGF	IMU-131 and ramucirumab plus paclitaxel	111 patients were stratified into two arms:	Median OS was 13.9 months (vaccine + chemotherapy) *vs*. 8.3 months (chemotherapy)	
			Arm 1 is 50 µg HER-Vaxx (IMU-131) + chemotherapy (ramucirumab plus paclitaxel) and Arm 2 is 50 µg HER-Vaxx (IMU-131) + 200 mg pembrolizumab every three weeks		
[51] Sundar et al., 2018/OTSGC-A24	VEGFR1	OTSGC-A24	24 patients were stratified into three arms:	40% of patients achieved SD.	No grades 4–5 AE were observed, and the most common lower grade AE decreased appetite, diarrhea, and myalgia
			Weekly vaccinated (1W)	Median PFS was 1.7 months [1.7 months (1W) *vs*. 1.6 months (2W) *vs*. 7.2 months (3W)]	
			Bi-weekly vaccinated (2W)		
			Tri-weekly vaccinated (3W)		

The KEYNOTE-059 was a global, phase II trial that evaluated the safety and efficacy of pembrolizumab in patients who previously received treatment with chemotherapy for gastric/gastroesophageal junction (G/GEJ) cancer (Table 2). These results guided the FDA approval of pembrolizumab for patients with recurrent or locally advanced G/GEJ adenocarcinoma with PD-L1 combined positive score (CPS) ≥ 1. Although the study comprised three cohorts of patients who had previously received different lines of therapy, the authors limited the scope of their article to the analysis of cohort 1 patients (who had been subjected to two or three prior therapies). 259 patients received pembrolizumab as monotherapy for two years, and tumor response was monitored at constant intervals during treatment. Results showed a complete response (CR) of 2.3% and a partial response (PR) of 9.3%. Moreover, 16.2% of the patients demonstrated stable disease (SD) and in 56% the tumor progressed, causing the treatment to be ceased. The median overall survival (OS) was 5.6 months, and the median progression-free survival (PFS) was 2 months. PD-L1 positive (CPS ≥ 1) tumors (57.1%) showed significantly better results (ORR = 15.5% with 2% CR) than PD-L1 negative tumors (42.1%) (ORR = 6.4% with 2.4% CR). Pembrolizumab was also more effective in patients who received it as third-line therapy instead of fourth, showing an ORR of 16.4% *vs*. 6.4%, respectively [31]. PD-L1 expression and microsatellite instability (MSI) status were two predictive factors for the efficacy of this treatment, as higher ORR was observed in patients with PD-L1 positive tumors and MSI-high tumors [31]. Treatment-related AE of grades 3–5 were reported in 17.8% of patients, the most common being fatigue, pruritus, and rash [31].

The KEYNOTE-158 is a phase II study conducted in patients with mutations in DNA mismatch repair genes and high microsatellite instability (MSI-H/dMMR) phenotype who have undergone first-line treatment with no success (Table 2). The dMMR phenotype can be associated with hereditary syndromes, such as Lynch syndrome, or in sporadic tumors MSI-H tumors have mutation rates 10 to 100-fold higher than MSI-deficient tumors, suggesting that they may be more immunogenic to the immune system, and consequently respond better to immunotherapy; Although this study included twenty-seven types of solid tumors, 24 participants (10.3%) had GC; all the patients were treated with pembrolizumab, and the results show a 9.9% CR, a 24.5% partial response (PR) and a 34.3% ORR. Complete responses were most frequently observed in GC; this tumor also showed an ORR of 45.8% and a median PFS of 11 months, one of the best results obtained. Regarding safety, grade 3–5 side effects occurred in 14.6% of patients, the most common being fatigue, pruritus, and diarrhea [32]. A more recent study described an updated analysis of the KEYNOTE-158 study, involving 351 patients with multiple types of solid tumors with dMMR/MSI-H phenotype. GC was one of the most prevalent in the study population, corresponding to 14.5% of all patients. Of these patients, 321 completed the study, 42 (13%) of which had GC; the ORR was 30.8%, similar to the previous study. The median duration of response (DOR) was lengthy at 47.5 months, the median PFS was 3.5 months and the median OS was 20.1 months [33].

The KEYNOTE-180 study is a phase II that focused primarily on advanced/metastatic esophageal adenocarcinoma or esophageal squamous cell carcinoma but also included patients with GEJ cancer that were resistant to two or more previous treatment lines (Table 2). This study evaluated patients irrespective of their PD-L1 status, however, PD-L1 status was assessed in all patient’s pre-treatment, and subgroup analyses were performed based on PD-L1 positive and negative tumors. As such, 121 patients were treated with pembrolizumab. The ORR for all patients was 9.9%, more specifically, patients with adenocarcinoma showed a 5.2% ORR; subgroup analysis showed that ORR was 2-fold higher in PD-L1 positive tumors (PD-L1 CPS of 10 or higher) (13.8%) compared to PD-L1 negative tumors (6.3%). 12.4% of patients experienced grade 3–5 treatment-related AE, namely hypothyroidism and pneumonitis [34,35].

The CheckMate-032 is a phase I/II clinical trial that evaluated the safety and efficacy of nivolumab monotherapy (PD-1 inhibitor), as well as nivolumab in combination with ipilimumab (CTLA-4 inhibitor), in patients with locally advanced or metastatic gastric, esophageal, or gastroesophageal junction cancer non-responsive to chemotherapy (Table 2). Janjigian et al. [36], following preclinical models (which have shown the synergistic effects of dual nivolumab and ipilimumab administration) and the ATTRACTION-2 trial, a placebo-controlled phase III trial that proved the nivolumab positive effects in patients survival, leading to the approval of nivolumab as second-line treatment for patients in Japan [36]. Of the 160 participating patients, three groups were formed which received nivolumab monotherapy (NIVO3) or combination therapy of nivolumab and ipilimumab (NIVO1+IPI3 and NIVO3+IPI1). Outcomes showed that the best ORR and PFS results corresponded to the combination group NIVO1+IPI3, being 24%, and 17%, respectively; also, the OS in this cohort was 6.9 months. The NIVO3+IPI1 group showed the worst results. However, the clinical advantage observed in the NIVO1+IPI3 group was associated with a higher occurrence of grade 3/4 AE of 47% *vs*. 17% (NIVO3) and 27% (NIVO3+IPI1). The most common AE were diarrhea and liver enzyme level elevation. Due to the higher ORR and OS rates observed in the NIVO1+IPI3 group [36], this arm was chosen for further evaluation in a phase III clinical trial, the CheckMate-649.

Janjigian et al. conducted CheckMate-649, a randomized, multicenter, phase III trial that aimed to assess the efficacy of combining nivolumab with chemotherapy *vs*. chemotherapy in patients with advanced, previously untreated, Human Epidermal growth factor Receptor-type 2 (HER-2)-negative G/GEJ cancer or esophageal adenocarcinoma (Table 2). Of 1549 patients, two cohorts were randomly formed (nivolumab plus chemotherapy *vs*. chemotherapy). Only patients with a PD-L1 CPS of 5 or higher were included in the study suggesting that PD-L1 CPS might be a better predictor for the efficacy of anti-PD-L1 treatment [37].

The median OS was slightly better for the nivolumab plus chemotherapy group (13.1 months) than the chemotherapy group (11.1 months), corresponding to a 29% reduction in risk of death in comparison to chemotherapy and a 3.3-month improvement in median OS (14.4 months *vs*. 11.1 months). The immunotherapy also provided superior PFS in patients with a PD-L1 CPS ≥ 5, resulting in a 32% reduction in the risk of disease progression or death *vs*. chemotherapy. Although the study found that nivolumab plus chemotherapy was associated with a significant improvement in OS for patients with higher PD-L1 CPS compared to chemotherapy alone, no improvement was observed in OS or PFS for patients with lower scores. Additionally, the treatment proved to be particularly effective in MSI-H and microsatellite-stable tumors, consistent with other studies that show a positive correlation between these two molecular characteristics and a better response to immunotherapy. Grades 3–4 AE were more frequent in the combination therapy group (59%) than in the chemotherapy group (44%), the more common being nausea, diarrhea, and peripheral neuropathy [37].

The KEYNOTE-811 is a global phase III study that aimed to evaluate the efficacy of adding pembrolizumab to trastuzumab and chemotherapy in patients with HER-2-positive advanced (unresectable or metastatic) G/GEJ cancer (Table 2). The pool of 433 patients was divided into two groups, that would either receive pembrolizumab or a placebo (control group) in addition to trastuzumab and chemotherapy [38].

In the pembrolizumab group, 11.3% of patients had a CR and 63.2% had a PR compared to the control group, with a CR and PR of 3.1% and 48.9%, respectively. This resulted in an ORR of 74.4% (pembrolizumab) *vs*. 51.9% (placebo). In the pembrolizumab group, about 70% of responses lasted over 6 months and 58% over 9 months, demonstrating the durability of this treatment. In the treated population, 57% of patients had grades 3–4 AE; the most frequently observed AE were diarrhea, nausea, and anemia. However, immune-mediated reactions were significantly more frequent in the pembrolizumab group (33.6%) compared to the placebo group (20.8%) [38].

CP-MGAH22–05 is a single-arm phase Ib/II study that evaluated the safety and efficacy of margetuximab (mAb against HER-2 receptor) combined with pembrolizumab in patients with advanced/metastatic, HER-2-positive gastro-esophageal adenocarcinoma which had progressed after at least one line of previous therapy (Table 2). Margetuximab can be used primarily to treat HER-2 expressing breast cancer or, in combination with pembrolizumab, in gastro-esophageal adenocarcinoma. Compared to trastuzumab, margetuximab has been modified to have a higher affinity to HER-2 and improve the anti-tumor immune response. These characteristics have shown an improvement in the DOR and PFS concerning to trastuzumab [39].

In the CP-MGAH22-05 study, the patients received margetuximab intravenously after pembrolizumab infusion (Table 2). Phase Ib was a dose-escalation phase that followed a 3 + 3 design starting with a small number of patients (usually three) and if the treatment is tolerated by patients, the next three patients will receive a higher dose, until a maximum tolerated dose is reached. Phase II included the administration of margetuximab (15 mg/kg) plus pembrolizumab (200 mg intravenously every three weeks) [39]. The results revealed an ORR of 18.48%, a median PFS of 2.73 months, and a promising disease control rate (DCR) of 53%. The median OS was 12.48 months. Tumors positive for PD-L1 and HER-2 expression showed significantly higher ORR and DCR values compared to tumors that do not express these molecules. After the treatment, 77% of patients demonstrated increased T-cell-mediated immune responses against several HER-2 antigens. Grade 3 or greater treatment-related AE occurred in 15% of patients (pembrolizumab) *vs*. 35.1% (chemotherapy). The most common AE were fatigue, decreased appetite, and nausea [39].

The KEYNOTE-061 is a randomized, phase III trial that compared pembrolizumab to a chemotherapeutic drug, paclitaxel, in PD-L1 positive metastatic or unresectable G/GEJ tumors that progressed after first-line treatment (Table 2). Among the 395 patients, 67% presented PD-L1 with a CPS of 1 or greater; these patients were allocated in a 1:1 proportion into two groups, one that would receive pembrolizumab and another that would be administered paclitaxel. The median OS for patients treated with pembrolizumab was 9.1 months, while those treated with paclitaxel had a median OS of 8.3 months, however, the difference was not meaningful. Regarding median PFS, pembrolizumab was 1.5 months and 4.1 months for paclitaxel. Nonetheless, patients treated with pembrolizumab had more lasting responses, with a median DOR of 18 months *vs*. 5.2 months for paclitaxel [35,40]. The study showed that pembrolizumab did not prolong OS compared to paclitaxel in patients with PD-L1 CPS ≥ 1 G/GEJ cancer. Fuchs et al. [41] updated the results of this study in 2022 (Table 2) [40]. The study included patients with PD-L1 CPS scores ≥ 5 and ≥ 10. In the ≥ 5 population, the median OS was 10.4 months for the immunotherapy group compared to 8.3 months for the chemotherapy group; for the CPS ≥ 10 population, the median OS was 10.4 months and 8 months for pembrolizumab and paclitaxel, respectively [40]. Regarding PFS, pembrolizumab was associated with a shorter median PFS than paclitaxel in the CPS ≥ 1 population (1.5 months *vs*. 4.1 months), as shown in the primary study, however, there was no significant difference in these values for the CPS ≥ 5 population, as PFS was of 1.6 months for pembrolizumab and 4 months for paclitaxel [35,40]. As for the CPS ≥ 10 population, median PFS was the same in the chemotherapy group but was slightly better for pembrolizumab, reaching 2.7 months. The ORR was better associated with higher PD-L1 CPS, reaching 24.5% in the CPS ≥ 10 population compared to 16.3% in the CPS ≥ 1 population. Median DOR was longer in the pembrolizumab group regardless of CPS status, however, as ORR, DOR increased as PD-L1 CPS increased [41]. Grades 3–5 treatment-associated AE occurred in 15% of patients treated with pembrolizumab compared to 35.1% in paclitaxel-administered patients, and the most common AE was fatigue and anemia for the immunotherapy group [41].

The KEYNOTE-062 is a phase III trial that was initiated by Shitara et al. [42] based on the KEYNOTE-059 and KEYNOTE-061 trials, which had shown promising results, including OS and fewer toxic effects [42]. It is a global, randomized phase III trial to evaluate the efficacy and safety of pembrolizumab alone or in combination with chemotherapy, compared to chemotherapy alone as a treatment for advanced GC patients (Table 2). The study enrolled 763 patients with untreated, advanced, or metastatic G/GEJ cancer with a PD-L1 CPS ≥ 1 and negativity for HER-2; these patients were randomized into three groups that received pembrolizumab alone, pembrolizumab in combination with chemotherapy and chemotherapy alone [42]. Concerning OS pembrolizumab alone and chemotherapy were similarly effective. However, although pembrolizumab did not demonstrate an advantage over chemotherapy in patients with PL-D1 CPS ≥ 1, in patients with PD-L1 CPS ≥ 10 the pembrolizumab group showed a significant improvement in median OS compared to chemotherapy alone (17.4 and 10.8 months, respectively). Nonetheless, PFS, OR, and CR were lower in the pembrolizumab groups; the DOR was longer in these same groups, especially in patients with PD-L1 CPS ≥ 10. AE of grades 3–5 was observed in around 70% of patients in the chemotherapy groups, compared to around 54% in the pembrolizumab monotherapy group, with the most frequent being fatigue, nausea, and decreased appetite. Immune-mediated AE was more incident in the pembrolizumab groups [42].

The KEYNOTE-181 was performed in patients with advanced/metastatic esophageal adenocarcinoma (including adenocarcinoma of the GEJ) or esophageal squamous cell carcinoma (Table 2) [43]. A total of 628 patients were divided into two groups on a 1:1 basis, one group being administered pembrolizumab and another chemotherapy. Median OS was significantly higher in the pembrolizumab group (9.3 months) compared to the chemotherapy group (6.7 months), meeting the prespecified threshold to demonstrate the advantage of pembrolizumab over chemotherapy. Median PFS was shorter in the pembrolizumab group (2.1 months) compared to the chemotherapy group (3.4 months); however, the results for the patients with PD-L1-positive tumors (CPS ≥ 10), this difference was minimal, with 2.6 months and 3.0 months for pembrolizumab and chemotherapy, respectively [35,43]. Regarding patients with PD-L1-positive tumors OR was 21.5% for patients receiving pembrolizumab *vs*. 6.1% receiving chemotherapy, with a median DOR of 9.3 months and 7.7 months, respectively. In PD-L1-negative tumors, pembrolizumab did not show beneficial outcomes compared to chemotherapy. Grades 3–5 treatment-related AE was experienced by 18.2% of patients for pembrolizumab and 40.9% for chemotherapy [35,43].

In a phase Ia/b trial from 2020 called JVDJ, the safety and efficacy of ramucirumab, a fully humanized IgG1 monoclonal antibody targeting the extracellular domain of VEGF receptor 2 (VEGFR2), and durvalumab, a PD-L1 inhibitor, for patients with non-small cell lung cancer, G/GEJ adenocarcinoma and hepatocellular carcinoma (HCC) were studied (Table 2) [44]. In patients with G/GEJ cancer, ramucirumab increased CD8^+^ T-cell infiltration within the TME, which in turn enhanced pembrolizumab’s activity. During this study, 85 patients received treatment with ramucirumab and durvalumab. Specifically in the G/GEJ patients’ group, 21% of patients had confirmed PR to treatment, as revealed by a reduction in tumor size. All responders, apart from one, had microsatellite-stable tumors and a PD-L1 CPS ≥ 25. Median DOR was long, reaching 15.4 months, and PFS was 2.6 months. The median OS was 12.4 months. Note that patients with high PD-L1 expression experienced the best results regarding tumor size reduction [44].

The JAVELIN Solid Tumor Trial was a phase I clinical trial with advanced/metastatic G/GEJ cancer patients who had undergone first-line chemotherapeutic treatment and were administered with avelumab (anti PD-L1) (Table 2) [45]. The patients were split into two groups: the second-line (2L) subgroup, who received chemotherapy over 28 days before the study began; and the first-line maintenance (1L-mn) subgroup, who recently finished first-line chemotherapy (within 28 days) and were continued with maintenance treatment throughout the trial. In two-week intervals, all patients received avelumab (an anti-PD-L1 mAb) after antihistaminic and anesthetic administration. The study enrolled a total of 150 patients (90 in the 1L-mn subgroup and 60 in the 2L subgroup) [45]. For the 1L-mn subgroup, the ORR was 6.7%; a CR in 2.2% and a PR in 4.4% were observed, with a DCR of 56.7% since 50% of patients in this subgroup only achieved SD status. Because an anti-PD-L1 mAb was used, better ORR was observed in the PD-L1 positive tumors (7.7% *vs*. 3.9%). The median DOR showed the remarkable durability of the treatment, reaching 21.4 months. The median PFS was 2.8 months, and the median OS was 11.1 months. Interestingly, the best results were obtained in Asian patients compared to other ethnicities [45]. Regarding the 2L subgroup, the ORR was 6.7% (similar to 1L-nm) but the DCR was lower, at 28.3%. The median DOR, however, was significantly lower, only 3.5 months. Median PFS was 1.4 months and the median OS was 6.6 months. 8.7% of patients experienced grade 3 or higher treatment-related AE at similar rates between subgroups, the most reported being fatigue, anemia, and asthenia [45].

In another study from 2023, Zhang et al. [46] aimed to investigate the safety and efficacy of apatinib (a tyrosine kinase inhibitor) and sintilimab (anti-PD-1 mAb) in combination with chemotherapy as a second- or third-line treatment for patients with previously treated and non-responsive advanced G/GEJ adenocarcinoma (Table 2). This was a prospective, single-arm, phase II trial [46]. The study involved 30 patients (2 patients were excluded due to incomplete data). Of the remaining 28 patients, 15 (53.5%) had a PR, 8 (28.5%) had SD, and 5 (17.8%) had progressive disease (PD) after treatment. The ORR was 53.6%, and the DCR was 82.1%. The median DOR was 8.8 months and the median PFS was 8.5 months; finally, the median OS was 12.5 months [46]. Additionally, several prognostic factors were analyzed. Patients with liver metastasis had a significantly higher ORR and longer median PFS and OS compared to those without liver metastasis. Also, patients with intestinal-type tumors showed a higher ORR and longer median PFS compared to those with diffuse/mixed tumors. Grades 3–4 AE was observed in 49.8% of patients, the most common being neutropenia and leukopenia [46].

The JACOB was a placebo-controlled, phase III trial that aimed to investigate the efficacy and safety of a treatment regimen consisting of pertuzumab and trastuzumab (both anti-HER-2 mAb) combined with chemotherapy in patients with untreated HER-2-positive, metastatic G/GEJ cancer (Table 2) [47]. The study enrolled 780 patients who were randomized in a 1:1 proportion into two arms–one would receive pertuzumab and another a placebo, in combination with trastuzumab and chemotherapy [47]. The pertuzumab group showed a median OS of 18.1 months and a median PFS of 8.5 months. Both endpoint values were lower, corresponding in the placebo group to 14.2 and 7.2 months, respectively. Median DOR was also higher in the pertuzumab group, reaching 10.2 months *vs*. 8.4 months for the placebo. Grades 3–5 AE were experienced by 80.5% and 74.2% of patients, respectively, in the pertuzumab and placebo groups [47].

In a phase I study published in 2018, Ishikawa et al. [48] assessed the effects of ACT in combination with mAb plus chemotherapy, The patients were treated with expanded Natural Killer (NK) cells and trastuzumab or cetuximab (an anti-epidermal growth factor receptor, or EGFR, mAb) based chemotherapy (Table 2) [48]. The 9 patients included in the study had gastric (33.3%) or colorectal (66.7%) carcinoma. The study followed a 3 plus 3 design, where patients received specific doses of chemotherapy drugs and mAb, followed by intravenous injection of expanded NK cells every three weeks for a total of three cycles (nine weeks) [48]. Response to treatment was evaluated using computer tomography. In six patients who had target lesions, three showed a decrease in the lesions’ diameters. Four patients showed SD and two demonstrated PD, with 66.7% DCR. The study concluded that toxicity did not increase when combining autologous expanded NK cells to mAb treatment plus chemotherapy, as AE had similar rates to chemotherapy in monotherapy. The most common AE were anemia thrombocytopenia and fatigue [48].

Qi et al. [49] published in 2022 the interim results of a phase I trial that assessed the activity of CAR-T cells specific to Claudin18.2 in gastrointestinal cancers; zolbetuximab is a mAb targeting CLDN18.2 which is currently being developed and has shown a 9% ORR in CLDN18.2-positive G/GEJ cancer patients that were non-responsive to at least one prior therapy line (Table 2) [49]. Promising results were obtained combining zolbetuximab with different chemotherapies; the results showed improved ORR and DOR. Furthermore, this study aimed to explore the anti-tumor effect of CLDN18.2 CAR-T cells in G/GEJ cancer patients, using CT041, a genetically engineered autologous T-cell therapy that expresses the CLDN18.2 targeting CAR, following preclinical data that demonstrated the anti-tumor effects in GC patients [49]. A total of 49 patients were infused with CT041, however, this interim analysis included only the first 37 patients who completed 12 weeks of safety assessment. For all the patients, the ORR was 48.6%, the DCR was 73%, the median PFS was 3.7 months, and the OS after 6 months was 80.1%. Comparatively, for the GC patient’s cohort, the ORR and the DCR increased to 57.1% and 75%, respectively; both median PFS and OS in 6 months were slightly better at 4.2 months and 81.2%, respectively [49]. Subgroup analysis showed that CT041 showed an ORR of 40% or higher in most subgroups, particularly in patients who had previously undergone and failed anti-PD-1/PD-L1 mAb and/or taxane treatment; additionally, ORR was higher in patients with Lauren intestinal type than other subtypes. Of note, at the time this interim analysis was conducted, the study was still ongoing [49].

The Ede et al.’s study [50] described the preclinical and clinical studies that have been conducted to test the safety and efficacy of a cancer vaccine, the HER-vaxx (IMU-131), in the treatment of HER-2-expressing cancers (such as GC) (Table 2) [50]. HER-vaxx is a B cell cancer vaccine to treat advanced G/GEJ adenocarcinoma. It contains a substance called P467-CRM197, constituted of three peptides from the Her-2 protein (polyclonal vaccine); these are highly immunogenic and can activate B-cells to produce antibodies against HER-2, which is often overexpressed by GC cells (10%–20% of cases), triggering an immune response against them. In GC patients, resistance to immunotherapy such as trastuzumab has been observed through a loss in HER-2 expression after treatment, accompanied by an increase in PD-L1 expression, which leads to immune escape and tumor progression [50].

Furthermore, HERIZON (IMU-ACS-001) is a clinical trial program that includes a phase Ib and a phase II clinical trial and aims to investigate the safety and efficacy of HER-vaxx for the treatment of G/GEJ cancer patients expressing HER-2 [50]. The phase Ib trial assessed the safety and immunogenicity of the cancer vaccine in 14 patients who were administered the vaccine on days 0, 14, 35, and 42 days; No significant adverse reactions occurred and the vaccination dose of 50 μg was sufficient for inducing the consistent production of high levels of antibodies; the titters of these antibodies correlated with a positive clinical response, and this dose was chosen for further investigation [50].

The phase II trial is a larger, randomized controlled trial designed to evaluate the efficacy and safety of HER-Vaxx in combination with chemotherapy. The study enrolled 111 patients who were randomly distributed into a group that would be administered HER-vaxx plus chemotherapy and another group that would receive chemotherapy alone [50]. Although the trial is still ongoing, interim results have been published and they showed significant OS benefits to the combination therapy, as median OS was 13.9 months for the HER-vaxx plus chemotherapy group *vs*. 8.3 months for the chemotherapy group; additionally, no additional toxicity was observed when adding the vaccine [50].

Finally, the next HORIZON is a proposed study that compares the use of HER-vaxx combined with chemotherapy and HER-vaxx plus pembrolizumab in patients whose G/GEJ tumors have progressed after treatment with trastuzumab [50].

A phase I/Ib study was conducted to analyze the safety of the OTSGC-A24 combined peptide vaccine in patients with advanced GC, aiming to determine the optimal dose and vaccine administration schedule, as well as its potential to induce good clinical outcomes (Table 2) [51]. The study enrolled patients with locally advanced/metastatic GC, and three cohorts of three patients were formed, which received a set dose of the vaccine at different intervals–one group was vaccinated weekly, another bi-weekly, and another tri-weekly; if an immune response occurred in two of the three patients composing group, that group would be expanded to ten patients to better assess immune response rates. The occurrence of an immune response was evaluated by measuring the cytotoxic T lymphocyte (CTL) response using an ELISPOT assay [51]. A total of 24 patients were treated but only 20 were analyzed for efficacy; SD was the best observed outcome, occurring in 40% of patients, and median PFS was 1.7 months, however, singular cohort analysis showed a significantly higher PFS in the tri-weekly vaccinated cohort (7.2 months) *vs*. 1.7 months (weekly vaccinated cohort) and 1.6 months (bi-weekly vaccinated cohort). The median OS was 5.7 months, with the best OS results being observed in the tri-weekly cohort [51].

## Discussion

GC tumors are characterized by a rich and diverse immune landscape suggesting immunotherapy as a promising treatment modality for this malignancy. In this section, we discuss and explore immunotherapy and its implications for clinical practice.

The KEYNOTE trials have been pivotal for evaluating the role of ICI in GC. KEYNOTE-012 proved the effectiveness of pembrolizumab in pretreated patients with advanced/metastatic G/GEJ cancer, as it demonstrated durable responses paired with manageable toxicity, thus establishing pembrolizumab as a viable treatment option [35]. Pembrolizumab acts by binding to PD-1, a receptor expressed on the surface of T-cells that, when activated by its ligands, inhibits T-cell-mediated responses. Pembrolizumab blocks the interaction between PD-1 and its ligands, restoring immune responses against cancer cells [52].

Although KEYNOTE-012 was a pivotal study and one of the first to investigate pembrolizumab in GC, it had notable limitations, such as its relatively small sample size and single-arm design, which may restrict the applicability of the findings [30]. Also, the trial focuses on objective response rates and safety endpoints, without long-term survival data or detailed biomarker analyses, which presents challenges in assessing pembrolizumab’s comparative efficacy and defining its role in treatment. Larger randomized trials are warranted to validate these findings.

To address these limitations, KEYNOTE-059 emerged as a crucial follow-up study that further investigated pembrolizumab, using a larger cohort of patients, and once again demonstrating its efficacy for GC patients. The best results were obtained in patients with PD-L1-positive advanced/metastatic G/GEJ cancer, who showed more durable responses and better overall outcomes [31]. While Keynote-059 provided valuable insights into the efficacy of pembrolizumab as a second line for advanced GC, it had limitations once the pembrolizumab was used without comparison to standard chemotherapy. To address this gap and better understand the comparative effectiveness of both treatments, further studies were warranted to directly compare pembrolizumab with chemotherapy.

Keynote-061 included two separate groups of patients receiving either pembrolizumab or chemotherapy and compared the outcomes. The study demonstrated improved OS in pembrolizumab compared to chemotherapy alone, especially in patients with high PD-L1 CPS. Although PFS was lower for the immunotherapy groups, the DOR was significantly higher for these patients compared to those undergoing chemotherapy. The lower PFS rates observed in the immunotherapy groups may stem from two main factors: firstly, immunotherapy often induces a phenomenon known as pseudo-progression, which temporarily increases tumor size before eventual shrinkage, mimicking disease progression and potentially skewing PFS assessments; secondly, over time, some tumors may develop resistance to immunotherapy, compromising its long-term efficacy [45].

The study also points out how patient characteristics regarding PD-L1 expression may influence treatment response, with subgroup analysis accentuating the differences in response based on this factor [40]. Similarly, in the JAVELIN solid group trial, the best results were obtained in Asian ethnicity patients, emphasizing the need for tailoring treatment strategies to each patient’s unique characteristics [45,46,49]. However, this study design also has limitations, as differences in patient characteristics between treatment arms can lead to bias in the results.

As such, KEYNOTE-062 explored pembrolizumab alone or in combination with chemotherapy and compared the results in patients treated with chemotherapy alone. Additionally, KEYNOTE-180 investigated pembrolizumab in combination with standard-of-care chemotherapy in a first-line treatment setting for advanced/GEJ cancer, revealing improved outcomes for patients receiving pembrolizumab in comparison to chemotherapy alone. Interestingly, these studies showed that patients still experienced a favourable safety and efficacy profile with pembrolizumab even after being exposed to the high toxicity associated with previous lines of therapy [34,42]. This highlights the value of exploring synergistic treatment approaches to maximize positive clinical outcomes.

The advances in GC profiling allowed the identification of molecular subtypes, that established the foundation for personalized therapy in GC. Challenges in molecular targeted therapy for gastric cancer: considerations for efficacy and safety [49,50]. KEYNOTE-158 specifically focused on patients of the MSI-H/dMMR phenotype with advanced/metastatic G/GEJ adenocarcinoma, who had undergone at least two previous lines of chemotherapy unsuccessfully; in this study, pembrolizumab demonstrated clinically relevant ORR coupled with an acceptable safety profile [53]. dMMR tumors generally present MSI-H due to the accumulation of several somatic mutations, expressing more neoantigens, being more immunogenic, and activating the upregulation of immune checkpoint proteins.

KEYNOTE-811 assessed the effects of adding of pembrolizumab to trastuzumab and chemotherapy for HER-2 positive advanced G/GEJ cancer, resulting in improved OS and PFS [33]. Trastuzumab binds to the extracellular domain of HER-2, thereby blocking a series of pathways that participate in cell growth and survival. Trastuzumab also activates the immune system through antibody-dependent cellular cytotoxicity (ADCC) and antibody-dependent cellular phagocytosis (ADCP), recruiting phagocytic immune cells to recognize and destroy HER-2-positive tumor cells [49,50]. Additionally, CP-MGAH22-05 investigated pembrolizumab and margetuximab combination therapy in advanced, HER-2-positive, G/GEJ cancer, showing encouraging response rates. Tumors positive for PD-L1 and HER-2 showed significantly higher ORR and DCR values compared to other tumor types [34,51,54].

These three studies highlighted once more, the importance of the molecular subtype in predicting clinical outcomes for GC patients, using combination therapies for each specific molecular subtype of GC. The implementation of this approach in clinical practice promoted personalized treatment approaches, potentially leading to better outcomes.

The CheckMate trials continued the investigation on ICI immunotherapy but instead focused on nivolumab, another anti-PD-1 antibody. Comparably to KEYNOTE-012, CheckMate-032 evaluated the effects of nivolumab monotherapy in heavily pretreated patients with metastatic G/GEJ cancer, resulting in encouraging responses with a manageable safety profile. Additionally, the study included the combination of nivolumab with ipilimumab, providing evidence that combining these two ICI demonstrated more positive clinical outcomes and durable anti-tumor activity while maintaining manageable safety, as observed by the higher ORR in the combination group [31].

CTLA-4 is expressed on the surface of T cells and downregulates their activity by competing against CD28, a co-stimulatory receptor, to bind with its ligands—CD80 and CD86. CD80 and CD86 are present on the surface of antigen-presenting cells and, when they bind to the CD28 on T-cells, they trigger T-cell activation. However, CTLA-4 affinity for these ligands is higher than CD28, therefore cancer cells tend to overexpress CTLA-4, consequently inhibiting T-cell-mediated immune responses [17,31,51].

Although no differences were observed in OS, the potential presence of more patients with MSI-H and PD-L1 positive tumors in the monotherapy group compared to the combination therapy could explain these results. Once the presence of MSI-H is associated with a higher expression of neoantigens, translating to a more immunogenic tumor, this effect might contribute to create a bias on the observed results. On the other hand, the combination therapy showed comparable clinical activity to nivolumab monotherapy, and with a higher incidence of AE, potentially limiting its overall benefit [31].

The CheckMate-649 trial investigated nivolumab combined with chemotherapy as a first-line treatment for advanced/metastatic G/GEJ cancer. The study reported that OS and PFS results for the combination therapy were significantly improved compared to chemotherapy alone, establishing nivolumab plus chemotherapy as a possible new standard-of-care treatment for this patient population [32]. Although the trials provide strong evidence for the safety and efficacy of ICI in GC treatment, several other studies have delved into less explored ICI and other immunotherapy approaches.

The Claudin18.2 study highlighted the potential of this antigen as an immunotherapy target in GC, revealing encouraging clinical responses in patients with Claudin18.2-positive tumors when using both an ICI (zolbetuximab) and CLDN18.2 CAR-T cell therapy [45,49].

The JVDJ trial combined ramucirumab and durvalumab for patients with advanced G/GEJ adenocarcinoma. The promising results for OS and PFS, paired with the lengthy DOR, demonstrated the potential of this combination [40]. Ramucirumab binds to the VEGFR2 molecule, which plays a pivotal role in tumor angiogenesis. By binding to the extracellular domain of this molecule, the interaction between VEGFR2 and its ligands is inhibited, blocking some signaling pathways involved in angiogenesis [44]. This anti-angiogenic effect helps to limit tumoral nutrient and oxygen supply, thereby restricting tumor growth. Additionally, the normalization of the abnormal vasculature within tumors improves drug delivery and enhances the efficacy of other therapies, like chemotherapy [55].

The Ishikawa et al. study explored the effects of ACT in combination with mAb and chemotherapy, demonstrating enhanced antitumor responses in the combination group, as well as prolonged survival in GC patients and a very high DCR [48].

Lastly, Ede et al.’s study explored the safety and immunogenicity of HER-vaxx, a cancer vaccine targeting HER-2, in patients with HER-2-positive G/GEJ cancer, by comparing it with chemotherapy. Results were promising, including significant OS benefits that were not associated with further toxicity compared to chemotherapy [50].

Combination therapies targeting multiple pathways essential for cancer cell survival can diminish the risk of treatment resistance due to mutation emergence. Recent studies have identified novel therapeutic targets and strategies for GC immunotherapy. Wang et al. [56] elucidated phosphatase and tensin homolog (PTEN) role as a barrier for cancer progression, suggesting it as a therapeutic target for GC treatment; since PTEN loss or inactivation is linked to increased tumor aggressiveness and poor clinical outcomes in GC, restoring its function using gene therapy emerged as a promising strategy to combat the tumor’s progression. Farkhondeh et al. proposed targeting Nrf2 since its inhibition in cancer cells seemed to enhance sensitivity to chemotherapy and reverse resistance to both anti-HER-2 drugs and various chemotherapy agents [15]. Moving forward, further exploration and clinical validation of these targets and strategies, as well as their combination with previously established immunotherapies, hold great potential to improve significantly patient outcomes.

**Figure 4:**
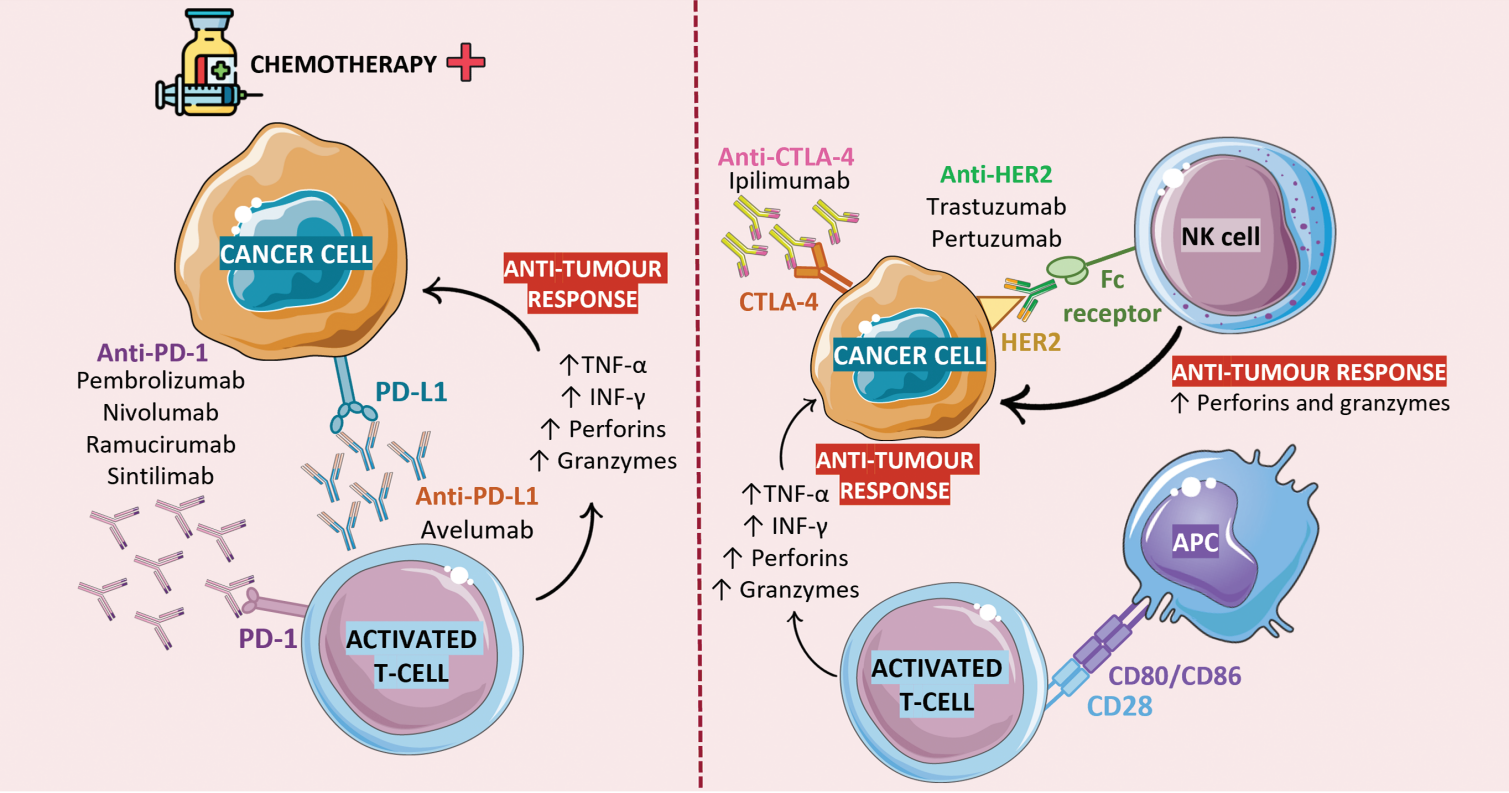
Immunotherapy in the treatment of patients with GC. To maintain the immune system in check and prevent the development of autoimmune diseases, T cells express inhibitor molecules on the surface: PD-1 and CTLA-4. From 30% to 50% of patients with GC overexpress the PD-1 ligand in their neoplastic cells. Upon its interaction with PD-L1, T cells’ immune and cytotoxic activity is suppressed and, consequently, tumor progression occurs. Likewise, the interaction of CTLA-4 with its ligands (CD80/CD86), which are expressed on the surface of antigen presenting cells (APC) prevents the proliferation and activity of T cells. Chemotherapy is currently the gold standard treatment for GC, and its combination with anti-PD-1 agents has been shown to improve immune response. Although several studies and clinical trials have proven the success of this approach as a form of treatment for many cancer types, a major roadblock to their widespread use in anti-cancer therapy is their dependence on pre-existing TIL to work. As such, patients with less immunogenic tumors or with a less permeable endothelial barrier (a layer of endothelial cells that line tumor blood vessels and regulate the transport of cells and substances into and out of the TME) may be non-responsive to this treatment approach. The combination of TIL therapy and ICI therapy could represent a workaround for this problem, as the expanded TIL would complement ICI action in building anti-tumor responses. Figure created using Biorender (https://biorender.com/, accessed on 21 November 2024).

The best results were obtained when using the anti-PD-1 mAb pembrolizumab. However, not all patients benefited the same way from this treatment modality. Furthermore, the most important influencing factor for variation in treatment response is undoubtedly the molecular subtypes of GC and biomarker expression, as we frequently observed the influence of this factor in predicting treatment response. Better results were consistently observed in higher PD-L1 expression cases and MSI-H/dMMR phenotype tumors. Prior treatment history also altered clinical responses, as patients who received one or two prior lines of chemotherapy demonstrated better outcomes when pembrolizumab treatment was administered. Regarding safety, the studies consistently reported manageable safety profiles, with AE being consistent with those already known to be associated with immunotherapy. Immune-related AE was frequently observed, especially with pembrolizumab treatment, reinforcing the importance of careful symptom monitoring and the development of management strategies. Fig. 4 summarizes these results.

Nevertheless, GC is an inherently heterogenous disease and coupled with dynamic TME and diverse molecular subtypes, poses a real challenge to the clinical implementation of immunotherapy on a widespread scale. Since each patient’s tumor is unique, immunotherapy, although promising, does not always show uniform effectiveness across various patient populations.

Acquired resistance to immunotherapy also poses a substantial obstacle in the clinical management of GC, as evidenced by the induction of long-lasting tumor responses followed by relapsing in a significant number of patients receiving checkpoint blockade therapy. This development of resistance over time renders immunotherapeutic agents ineffective for these patients. Multiple mechanisms can underline acquired resistance to immunotherapy, including loss of T cell function, downregulation of tumor antigen presentation, the emergence of escape mutation variants on cancer cells, and genetic alterations impacting the antigen-presenting system [57].

To overcome these challenges, it is crucial to understand the molecular mechanisms underlying these phenomena and devise targeted interventions to counteract them effectively. A promising strategy could involve combining ICI with complementary immunotherapeutic approaches. Adoptive T cell therapy presents a potent strategy to tackle the loss of T cell function, while cancer vaccines can effectively address the downregulation of tumor antigen presentation.

In this study, we explore the advances in immune-based therapeutic approaches in GC. There are some limitations. Firstly, for this systematic review, only one database was used. Despite the extensive coverage of peer-reviewed articles by PUBMED, when searching for relevant references, it is desirable to use multiple databases. Secondly, the difference between the number of patients involved in each study may have led to partial differences.

## Conclusion

Pembrolizumab emerged as the most efficient immunotherapeutic strategy in the context of GC, particularly in the subset of patients with the dMMR/MSI-H phenotype tumors and tumors expressing PD-L1. Pembrolizumab demonstrated the ability to induce durable anti-tumor responses and extend patients’ survival with advanced disease, transcending the outcomes achieved through standard therapies while maintaining a relatively good safety profile. However, it is important to acknowledge that ICI has been extensively studied in GC compared to cancer vaccines and ACT. As such, future research efforts should focus on exploring the potential of combination therapies involving pembrolizumab, as well as further investigating the use of other immunotherapeutic approaches. Immunotherapy challenges in the clinical set are treatment resistance, as well as combination therapy optimization, which is crucial for therapeutic landscape improvement for GC patients.

## Supplementary Materials





## Data Availability

Not applicable.

## Abbreviations

ACT: Adoptive Cell Therapy
AE: Adverse Event
CAR: Chimeric Antigen Receptor
CPS: Combined Positive Score
CR: Complete Response
CTLA-4: Cytotoxic T-lymphocyte-associated antigen 4
dMMR: Deficient in DNA Mismatch Repair
DOR: Duration of Response
EGFR: Epidermal Growth Factor Receptor
EMT: Epithelial to Mesenchymal Transition
FDA: Food and Drug Administration
G/GEJ: Gastric/Gastroesophageal Junction
HBV: Hepatitis B Virus
HCC: Hepatocellular Carcinoma
HER-2: Human Epidermal Growth Factor Receptor-type 2
HPV: Human Papilloma Virus
IC: Immune Checkpoint
ICI: Immune Checkpoint Inhibitor
mAb: Monoclonal Antibody
MHC: Major Histocompatibility Complex
mRNA: Messenger RNA
MSI-H: Microsatellite Instability High
MSI-H/dMMR: Deficient in DNA Mismatch Repair and High Microsatellite Instability
NK: Natural Killer
ORR: Objective Response Rate
OS: Overall Survival
PD: Progressive Disease
PD-1: Programmed Cell Death 1
PD-L1: Programmed Death-Ligand 1
PFS: Progression-Free Survival
PR: Partial Response
SD: Stable Disease
TAA: Tumor Associated Antigen
TIL: Tumor Infiltrating Lymphocyte
TME: Tumor Microenvironment
TNM: Tumor, Node, Metastasis
VEGFR2: Vascular Endothelial Growth Factor Receptor 2
*vs*.: *versus*

## Supplementary Materials

The supplementary material is available online at https://doi.org/10.32604/or.2024.052207.
